# A systematic evaluation of the therapeutic potential of endogenous-ADAR editors in cancer prevention and treatment

**DOI:** 10.1093/narcan/zcaf016

**Published:** 2025-05-06

**Authors:** Rona Merdler-Rabinowicz, Ariel Dadush, Sumeet Patiyal, Padma Sheila Rajagopal, Gulzar N Daya, Shay Ben-Aroya, Alejandro A Schäffer, Eli Eisenberg, Eytan Ruppin, Erez Y Levanon

**Affiliations:** Cancer Data Science Lab, Center for Cancer Research, National Cancer Institute, National Institutes of Health, Bethesda, MD, 20892, United States; Mina and Everard Goodman Faculty of Life Sciences, Bar-Ilan University, Ramat Gan, 5290002, Israel; The Institute of Nanotechnology and Advanced Materials, Bar‐Ilan University, Ramat Gan, 5290002, Israel; Mina and Everard Goodman Faculty of Life Sciences, Bar-Ilan University, Ramat Gan, 5290002, Israel; The Institute of Nanotechnology and Advanced Materials, Bar‐Ilan University, Ramat Gan, 5290002, Israel; Cancer Data Science Lab, Center for Cancer Research, National Cancer Institute, National Institutes of Health, Bethesda, MD, 20892, United States; Cancer Data Science Lab, Center for Cancer Research, National Cancer Institute, National Institutes of Health, Bethesda, MD, 20892, United States; Cancer Data Science Lab, Center for Cancer Research, National Cancer Institute, National Institutes of Health, Bethesda, MD, 20892, United States; Mina and Everard Goodman Faculty of Life Sciences, Bar-Ilan University, Ramat Gan, 5290002, Israel; Cancer Data Science Lab, Center for Cancer Research, National Cancer Institute, National Institutes of Health, Bethesda, MD, 20892, United States; Raymond and Beverly Sackler School of Physics and Astronomy, Tel-Aviv University, Tel Aviv, 6997801, Israel; Cancer Data Science Lab, Center for Cancer Research, National Cancer Institute, National Institutes of Health, Bethesda, MD, 20892, United States; Mina and Everard Goodman Faculty of Life Sciences, Bar-Ilan University, Ramat Gan, 5290002, Israel; The Institute of Nanotechnology and Advanced Materials, Bar‐Ilan University, Ramat Gan, 5290002, Israel

## Abstract

Adenosine deaminases acting on RNA (ADAR) enzymes constitute a natural cellular mechanism that induces A-to-I(G) editing, introducing genetic changes at the RNA level. Recently, interest in the endogenous-ADAR editor has emerged for correcting genetic mutations, consisting of a programmed oligonucleotide that attracts the native ADAR, thereby offering opportunities for medical therapy. Here, we systematically chart the scope of cancer mutations that endogenous-ADAR can correct. First, analyzing germline single nucleotide variants in cancer predisposition genes, we find that endogenous-ADAR can revert a fifth of them, reducing the risk of cancer development later in life. Second, examining somatic mutations across various cancer types, we find that it has the potential to correct at least one driver mutation in over a third of the samples, suggesting a promising future treatment strategy. We also highlight key driver mutations that are amenable to endogenous-ADAR, and are thus of special clinical interest. As using endogenous-ADAR entails delivering relatively small payloads, the prospects of delivering endogenous-ADAR to various cancers seem promising. We expect that the large scope of correctable mutations that are systematically charted here for the first time will pave the way for a new era of cancer treatment options.

## Graphical Abstract

**Graphical Abstract:**
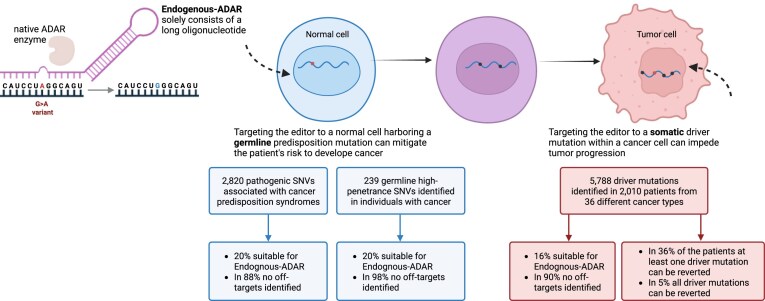

## Introduction

Base editing is an arsenal of techniques that enable the precise manipulation of the genome sequence by efficiently converting nucleotides. Initially explored for inherited diseases, base editing holds the potential to address various genetically driven disorders, as many inherited diseases arise from single pathogenic point mutations involving altered nucleotides, referred to as single nucleotide variants (SNVs) [1, 2]. In the context of cancer, the high mutational burden of tumors may appear to constrain the efficacy of such techniques. Yet, we propose that a novel technology utilizing endogenous RNA-editing enzymes [3] may prove beneficial.

Ongoing investigations have shown promising initial outcomes for several common genetic diseases [4]. The leading breakthrough demonstrated the restoration of the common *SERPINA1* variant, which causes the genetic disease α-1-antitrypsin deficiency, in nonhuman primates [5]. Following this success, the public company Wave Life Sciences has announced the first-ever worldwide dosing of two patients with this variant.

In addition to reverting common pathogenic variants, the precise programmability for specific sequences facilitates customization for rare genetic variants [6], tailoring them to individual patients within affordability and delivery constraints. This substantially broadens the applicability of endogenous RNA editing to address a wide range of genetic abnormalities. Here, we suggest that by targeting critical variants—germline predisposing mutations or somatic driver mutations—there is potential to exert a substantial impact in cancer. A compelling study was recently conducted in glioblastoma, wherein selective editing of the mutant *TERT* promoter using a DNA base editor led to preclinical benefit [7]. While the technology used in this case was not RNA editing, the study still gives hope for the potential of RNA editing.

The core of programmable RNA editing revolves around the design of a guide RNA (gRNA). The gRNA ensures target specificity through Watson–Crick base pairing to the desired region and also harnesses the native adenosine deaminases acting on RNA (ADAR), which is responsible for the extensive metazoan A-to-I editing [8], that is then read as G by the cellular machineries [9, 10]. As ADAR is naturally expressed at high levels in all body tissues, either the cytoplasmic isoform p150 or the nuclear isoform p110 [11], this method offers the advantage of a small molecular payload, in contrast to other base-editing methods. Delivering only the programmed chemically modified oligonucleotide to any target cell eliminates the need for an external viral vector or plasmid to insert the enzyme [12], thereby also reducing the immunogenic response to foreign components. Recent studies indicate that a guide consisting of merely 30–40 nucleotides can be sufficient, making this approach highly appealing [5, 13].

Unlike other base editors that can be tailored to target either a DNA sequence or an RNA sequence [14, 15], endogenous-ADAR is capable of changing only RNA sequences [3, 16]. The RNA-only limitation can be viewed as more cautious than DNA editing because editing the DNA sequence involves the risk of introducing irreversible errors into the DNA. Such errors may result from gRNA binding to undesired regions, leading to the unintentional conversion of distant nucleotides (off-target sites), or when the gRNA is correctly bound but the enzyme edits additional nucleotides in the vicinity of the targeted nucleotide (bystander edits) [17]. In contrast, targeting the RNA is considered safer, given that RNA molecules are constantly synthesized and degraded within cells [18]. The drawback of RNA editing is that this approach requires continuous and prolonged treatment, posing challenges for patients in adherence and feasibility. In addition, Clustered Regularly Interspaced Short Palindromic Repeats (CRISPR)-guided base editors were found to induce transcriptome-wide RNA errors [19, 20], including posing carcinogenic effects on cells [21, 22]. Another distinction lies in the spectrum of editable variants, as endogenous-ADAR operates exclusively on G > A variants at the RNA level. Yet, it is worth noting that because RNA editing may occur before splicing, it has the flexibility to target all gene regions, encompassing introns, excluding promoters. Since most RNA editing occurs shortly after transcription [23], it could correct potentially harmful variants before the nonsense-mediated decay (NMD) cellular mechanism is triggered. In fact, since gene expression and RNA editing are not directly correlated, RNA editing can effectively target the majority of variant types, with the exception of variants located in promoters that result in silent expression, which are relatively rare.

In the context of cancer, endogenous-ADAR present an additional noteworthy advantage; Expression levels of the natural ADAR enzymes (both ADAR1 and ADAR2) are rather high in most cancer types and the expression levels of the main editing enzyme, ADAR1, is known to be elevated in most cancer types [24], which may contribute to the efficacy of the treatment, as well as an increased selectivity to cancer versus non cancer cells.

Here, our objective is to explore systematically the potential of endogenous-ADAR for cancer prevention and treatment, considering its unique advantages and straightforward design for therapy. The first approach we explore focuses on cancer risk reduction/prevention, leveraging the existing application of endogenous-ADAR to genetic diseases. Inherited variants in cancer predisposition genes (CPGs) do not necessarily lead to cancer in infancy but rather are associated with increased lifetime risks of developing cancers [25]. Correcting these variants before cancer initiation holds the potential for preventive intervention. The second approach explores potential avenues for cancer treatment, utilizing endogenous-ADAR to correct driver mutations essential for tumor development and progression. We hypothesize that since only a small subset of the tumor’s mutations are considered drivers, directing the therapy toward key driver variants, central to cancer initiation and progression, holds the potential to improve a patient's trajectory. This reasoning is in line with current precision oncology therapies that mostly target a single mutation driver genes.

To evaluate the feasibility of this approach, we map the landscape of inherited cancer predisposition mutations and driver mutations that can be corrected via endogenous-ADAR across multiple cancer types. Leveraging endogenous-ADAR in this manner may introduce a new and powerful weapon to the arsenal of precision medicine techniques.

## Materials and methods

### Germline mutation data

We obtained (a snapshot of) the ClinVar database [26] from the UCSC Browser on 8 November 2021. At that time, the database contained 1 103 629 mutations, with 984 981 being SNVs. We excluded 147 genetic downstream and 1067 genetic upstream transcript variants, 237 mutations with no sequence alteration, 77 091 that had no molecular consequence information, 391 mutations that were mistakenly classified as SNVs and 2 052 mitochondrial mutations. This left us with 973 996 SNVs located in genes. Next, we filtered the 98 513 SNVs that were reported to be clinically pathogenic. We considered any phrase containing derivatives of the word “pathogenic” (e.g. “pathogenic”, “likely pathogenic”) to include these variants. In cases where there were conflicting interpretations of pathogenicity, we included the variant if at least one submitter reported it as pathogenic according to the ClinVar VCF file. We used the isoform “MANE SELECT” as presented in ClinVar and the reported coordinates (column OrigName) of each variant, to extract the RNA sequence from the CDS FASTA file that was downloaded from the UCSC genome browser (http://genome.ucsc.edu) [27] on 27 March 2022, as well as the reading frame of each variant (i.e. the 3-nucleotide codon sequence).

We utilized the 64 CPGs used in a test by PreventionGenetics and described in the Genetic Test Registry (GTR) curated by NCBI [28]. Out of the 98 513 SNVs examined, 8251 were found in these specified genes. We further filtered the variants, focusing only on those classified as causing Hereditary cancer-predisposing syndrome based on the classification provided by MedGen [29] (MedGen UID: 14 326, Concept ID: C0027672, Neoplastic Process). In total, our analysis included 2820 human pathogenic SNVs known to be associated with hereditary cancer syndromes and conditions in 40 different genes. For each variant, we extracted the DNA sequence using Bedtool getfasta (hg38 reference human genome).

In the second analysis, we used data curated by Srinivasan *et al.* [30], a study that investigated the context-specific role of germline pathogenicity in tumorigenesis from the MSK-IMPACT cohort of 17 152 adult patients with cancer who underwent prospective sequencing. To obtain the relevant data, we accessed their SignalDB website [30] and manually extracted the 450 high-penetrance germline mutations. In selecting only SNVs, this number was reduced to 248. In 9 cases, the cancer type was not specified, resulting in a final set of 239 SNVs that were included in our analysis.

For the off-target analysis we utilized the BLAT [31] program, searching for areas in the human genome that displayed substantial similarity to the 40 bases surrounding each variant. Different RNA editing methods use different lengths but the region that complements the guide is typically at most 40 nucleotides. We employed cautious parameters of 85% identity (varying this parameter from 75% to 95% did not have any discernible impact on the results) and a minimum alignment length of 20 nucleotides. For the bystander analysis, we examined the 20 bases surrounding each variant using the AlphaMissense prediction model [32], which evaluates the impact of all possible nucleotide changes on protein function. Changes that were defined by the model as “Pathogenic” or “Likely pathogenic” were counted. We utilized either the hg19 or hg38 version of the model, depending on the variant database under investigation.

### Driver mutations analysis

To evaluate the potential of endogenous-ADAR in reverting driver mutations, we acquired publicly available and controlled data from the PCAWG project on 3 May 2023. The dataset comprised 5788 driver mutations identified in 2010 patients across 36 distinct cancer types.

To compute the proportions of endogenous-ADAR targetable variants, we divided the number of editable variants by the total number of mutations in each specific cancer type. To determine the statistical significance of the editable proportions in each cancer type compared to the others, we employed the nonparametric Mann–Whitney *U* test, also known as the Mann–Whitney–Wilcoxon test. Additionally, we applied FDR multiple-testing correction to account for multiple comparisons and evaluate the statistical differences between the groups.

To determine the cumulative percentage of cells undergoing editing, we employed these probability equations when a single driver mutation edit is sufficient ($N$ is the number of driver mutations, $X$ is the probability of correcting one mutation in a given cell, $Y$ is the probability to correct at least $N$ mutations):


\begin{eqnarray*}
\begin{array}{@{}*{2}{c}@{}} {N = 1}&{\ Y = X}\\ {N = 2}&{Y = 1 - {{{\left( {1 - X} \right)}}^2}}\\ {N = 3}&{Y = 1 - {{{\left( {1 - X} \right)}}^3}} \end{array},
\end{eqnarray*}

and when two driver mutations edits are required:


\begin{eqnarray*}
\begin{array}{@{}*{2}{c}@{}} {N = 1}&{Y = 0}\\ {N = 2}&{Y = {{X}^2}}\\ {\begin{array}{@{}*{1}{c}@{}} {N = 3}\\ {N = 4} \end{array}}&{\begin{array}{@{}*{1}{c}@{}} {Y = 3{{{\mathrm{X}}}^2}\left( {1 - {\mathrm{X}}} \right) + {{{\mathrm{X}}}^3}}\\ {{\mathrm{Y}} = 6{{{\mathrm{X}}}^2}{{{\left( {1 - {\mathrm{X}}} \right)}}^2} + 4{{{\mathrm{X}}}^3}\left( {1 - {\mathrm{X}}} \right) + {{{\mathrm{X}}}^4}} \end{array}} \end{array}
\end{eqnarray*}

## Results

### Overview

First, we focus on variants within established pediatric CPGs to demonstrate the potential of reverting germline mutations, as correcting them offers the most direct potential benefit. Additionally, we examine pathogenic germline mutations associated with high penetrance cancer predisposition in adults. For each variant correctable by endogenous-ADAR we further investigate the likelihood of distant off-target sites and pathogenic bystander edits.

In the second approach, aimed at showcasing the potential of reverting somatic driver mutations, we analyze a database of oncologic patients across various cancer types, where driver mutations were reported for each individual. We evaluate the potential for addressing each driver mutation using endogenous-ADAR, which involves assessing the likelihood of off-target and bystander edits. Samples and different cancer types are ranked by potential for editability.

### Targeting cancer predisposition germline mutations

**Figure 1.**
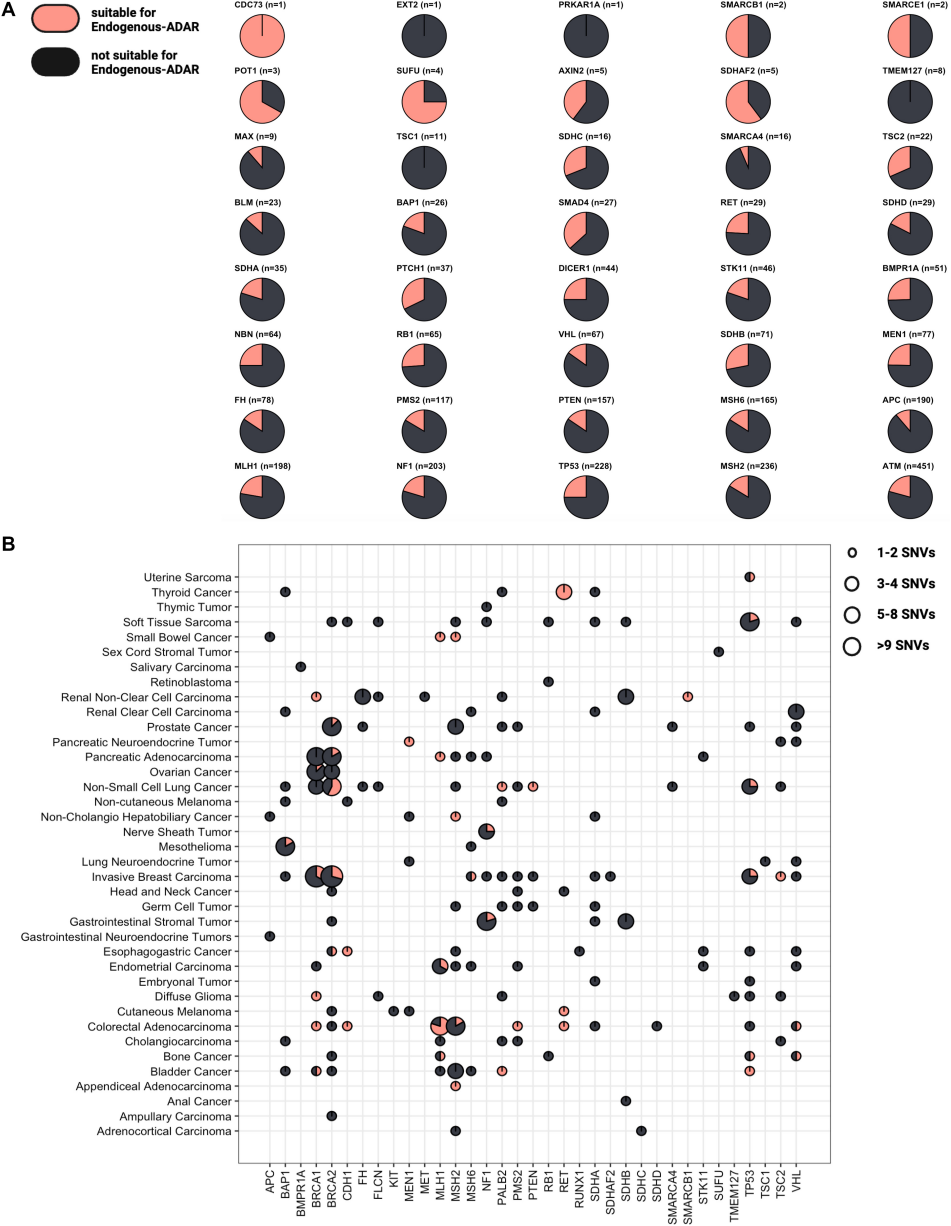
Distribution of targetable pathogenic germline mutations. (**A**) Distribution of established pathogenic germline mutations, categorized by CPGs: A total of 2820 SNVs linked to hereditary pediatric cancer risk syndromes are presented based on the respective genes. In parentheses: the total count of variants identified for each gene; in light/pink: SNVs suitable for endogenous-ADAR; in dark/black: SNVs not suitable for endogenous-ADAR. (**B**) Distribution of high-penetrance pathogenic germline mutations: A total of 239 germline SNVs obtained from the Memorial Sloan Kettering’s SIGNAL database, originating from sequencing of adult cancer patients, are presented by cancer subtype and gene. in light/pink: SNVs suitable for endogenous-ADAR; in dark/black: SNVs not suitable for endogenous-ADAR.

To assess the potential of endogenous-ADAR in targeting pathogenic germline mutations in CPGs, we implemented the following pipeline: We curated a set of 64 CPGs that are widely used in a test by PreventionGenetics for pediatric genetic testing due to their association with different genetic cancer clinical conditions [28] (Supplementary Table S1). We then extracted all pathogenic variants (Materials and methods) in these genes from ClinVar [26], and kept those classified as causing a “Neoplastic Syndrome” based on the classification provided by MedGen [29]. Our analysis ultimately included 2820 human pathogenic SNVs predicted to be associated with inherited pediatric cancer risk syndromes. Out of the 2820 curated SNVs, 566 (20%) were G > A variants, which are suitable targets for endogenous-ADAR (Fig. 1A). The G > A mismatch type occurs more frequently than expected because transition variants are more common than transversion variants, and the most frequent mismatch—C > T—results in a G > A conversion on the complementary DNA strand. Consequently, the prevalence of G > A pathogenic variants is notably higher than average.

### The risk of off-target effects and bystander edits is low

For precise on-target editing, meticulous gRNA design is essential to ensure binding to the targeted region and minimize the risk of off-target effects. This constraint limits the applicability in regions that exhibit high similarity to other genomic regions.

To identify potential off-target effects, we searched for areas in the human genome that displayed substantial similarity to the 40 bases surrounding each variant (Materials and methods), based on recent reports [13, 33, 34] highlighting the optimal guide length for ADAR-based techniques. Our findings indicate that 498/566 (88%) of the SNVs suitable for endogenous-ADAR do not have such off-target similar regions, suggesting that these variants are safer in terms of off-target effects.

Another concern is unintended bystander edits in close proximity to the target nucleotide. Even when the deaminase successfully approaches its target, it may inadvertently edit other nucleotides of the same type in the immediate vicinity. To explore this issue, we focused on the 20 bases surrounding the variant and quantified the number of adenosines in this region. Utilizing the recent prediction model of AlphaMissense [32], we further evaluated how many of them were predicted to cause a deleterious effect if editing occurs. We observed an average of 5.6 nearby adenosine (A) nucleotides available for potential editing. Out of these, an average of 0.7 was predicted to result in a likely-pathogenic effect when edited. In 347 SNVs (61%) no likely-pathogenic changes were predicted. These calculations may be used as a guideline for considering the adjacent nucleotides surrounding the target SNV while designing the guide. However, in practice, it is unlikely that all will be altered simultaneously. Also, A-to-I(G) editing does not have the potential to unintentionally introduce new stop codons (UAA, UAG, or UGA), making it a safer option in terms of bystander edits, compared to other base-editing methods. Encouragingly, a recent study demonstrated that the inclusion of an extra chemical compound alongside the gRNA significantly diminishes the occurrence of bystander edits [13]. Finally, it is worth mentioning again that even if a clinically deleterious mistake occurs, errors at the RNA level have only a transient effect.

### Frequency evaluation of variants amenable to endogenous-ADAR

When designing a therapy, considering its applicability to a larger patient population often favors common variants over rare ones. However, determining the frequency of a specific germline mutation in the general population is challenging due to genetic diversity and the rarity of most SNVs. The gnomAD database [35] compiles data from over 195 000 individuals worldwide, enabling approximate estimates of mutation frequency in the general population. gnomAD is exclusively based on adults, and individuals with known Mendelian diseases have been excluded. Consequently, the deleterious mutations documented are mostly heterozygous and found in genes associated with recessive diseases or dominant diseases with incomplete penetrance. Furthermore, gnomAD’s representation varies across populations, with an over-representation of European participants, leading to imprecise estimations of genetic variants and an underrepresentation of many communities, including those of African, Middle Eastern, and Oceanian descent [36]. Out of the 566 putative cancer-risk SNVs suitable for endogenous-ADAR editing, 102 were detected as present at least once in gnomAD (with an average frequency of 1.82 × 10^−5^). Variants that are present in gnomAD may be interesting candidates for future treatment consideration, but caution is warranted as discussed above. The two most frequent G > A SNVs in the dataset were *RET* c.1438G > A (p.Glu480Lys) and *TP53* c.91G > A (p.Val31Ile), with frequencies of 2.7 × 10^−4^ and 2.5 × 10^−4^, respectively. *ATM* (mutated in ataxia telangectasia) and *TP53* (mutated in Li-Fraumeni syndrome) stood out with the highest variant count in gnomAD, aligning with their well-established significance across a spectrum of cancers, along with the considerable length of *ATM*.

Supplementary Table S2 summarizes the pathogenic SNVs that are suitable for endogenous-ADAR, including added data we provided regarding the number of bystander edits and off-target hits. Note that in 313 out of the 567 variants, no off-target and no pathogenic bystander edit was observed, rendering them ideal targets in terms of safety.

Pediatric cancer predisposition syndromes present a potential use case for endogenous-ADAR. However, application in adult cancer risk populations may also be possible, albeit more challenging given the potential gene/environment mediators. Yet, delayed onset could present an advantage, providing a greater window of opportunity for intervention. We thus repeated the analysis using the high-penetrance gene list compiled by Memorial Sloan Kettering [30], comprised of 239 high-penetrance pathogenic germline SNVs associated with different cancer types in adults (Materials and methods). We found that 48/239 (20%) are suitable for endogenous-ADAR. The distribution of these SNVs across different cancer subtypes is illustrated in Fig. 1B. A noteworthy finding is that endogenous-ADAR can address all SNVs within the *RET* gene associated with thyroid cancer.

For 47 out of the 48 (98%) endogenous-ADAR targetable SNVs, no off-target sites were detected. Regarding potential bystander edits, we found an average of 5.2 A nucleotides per variant, of which 0.9 were predicted to be pathogenic. For 23 (48%) SNVs, no deleterious change is predicted. GnomAD reports frequency for 7 endogenous-ADAR targetable SNVs (with an average frequency of 1.02 × 10^−5^), of which 4 were identified in the *TP53* gene and 2 in the *MLH1* gene.

### Targeting cancer driver mutations as potential cancer treatments

**Figure 2.**
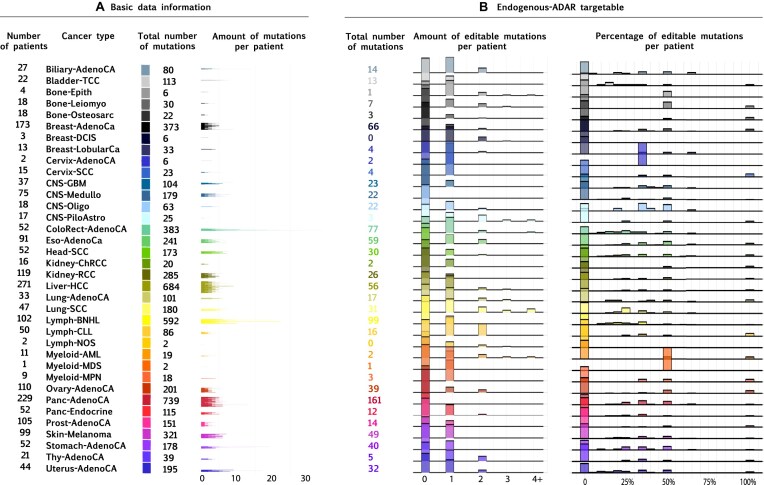
The potential of endogenous-ADAR in reverting cancer driver mutations. (**A**) Basic data information: Representation of the 2010 patients examined in our analysis, sourced from the pan-cancer analysis of whole genomes (PCAWG) project, categorized by their tumor subtypes, total count of identified driver mutations, and the number of driver mutations found per individual. (**B**) Driver mutations that are suitable for endogenous-ADAR: For each cancer type the absolute number and the percentage of variants per patient that can be reverted by endogenous-ADAR is shown.

Selecting optimal targets for correction in mature cancer tissue poses challenges due to the presence of numerous mutations, often unique to each individual. Consequently, a personalized approach involving the design of a tailored editor for each patient becomes necessary. Rather than targeting “passenger” mutations, it is reasonable to assume that targeting driver mutations will have more impact on clinically relevant tumor progression. For this analysis, we used the driver mutation list generated by the PCAWG project [37], which ranks variants in a genomic element based on recurrence, functional impact, and expected driver patterns. PCAWG identifies probable driver mutations by comparing the observed mutation burden to the background rate [37]. We analyzed 5788 driver mutations overall identified in 2010 patients from 36 different cancer types. The average number of driver mutations per patient was 2.9 (±2.4). Colorectal adenocarcinoma ranked highest on the list with an average of 7.4 (±7.1) driver mutations per patient. We found that 955/5788 (16%) of the driver mutations were suitable for endogenous-ADAR (Fig. 2). Of these variants, 857/955 (90%) had no detected off-target events, and on average, bystander edits extended over 4.7 nucleotides, with 1.9 predicted to be likely pathogenic. In 281 of the variants (29%) no likely-pathogenic changes were predicted. The comparison to the genome was performed utilizing a normal reference genome. Consequently, our calculations may not be entirely accurate when applied to a cancerous tissue, given the abnormal nature of the tumor genome that contains variations such as duplication events.

### Endogenous-ADAR correction is applicable to at least one-third of cancer cases

Given these results, we turned to calculate for each individual patient the percentage of driver mutations in her/his tumor that could be corrected. Notably, these percentages were conservatively determined by considering in the denominator all the driver mutations expressed by each patient, including the non-SNVs ones such as insertion/deletion mutations. We find that 729/2010 (36%) patients had at least one endogenous-ADAR targetable driver mutation; in 505/2010 (25%) patients at least 30% of the driver mutations were amenable to endogenous-ADAR; and in 310/2010 (15%) patients at least 50% of their mutations were treatable by endogenous-ADAR. Remarkably, in 91 (5%) patients, all driver mutations were suitable for endogenous-ADAR (81 patients had one driver mutation and 10 patients had two driver mutations). If reverting just one driver mutation per tumor has a clinical impact, our results suggest that more than a third of cancer patients may be suitable candidates for endogenous-ADAR therapy. This proportion exceeds the estimated number of patients with actionable mutations targeted by currently available cancer therapies [38].

### Different cancer types showed varied correction capacity

Different cancer types exhibit diverse distributions of mutation types, suggesting that the number of mutations correctable by endogenous-ADAR may vary among them. To rank cancer types based on their editability, we calculated the ratio of editable variants to the total number of mutations in each cancer type and evaluated the statistical significance of these editable proportions in comparison to other cancer types. Ten cancer types exhibited significantly higher editing potential by endogenous-ADAR, with the highest being pancreatic and liver (Table 1). Of interest, liver cancers are especially relevant, as current research in endogenous-ADAR focuses on the liver given the feasibility of delivering the therapy through direct intravenous injections [39].

**Table 1.** Cancer types that demonstrate significantly higher editing potential relative to others, when comparing cancer types in pairs. Ten such cancer types were detected for endogenous-ADAR

Cancer types	No. of patients involved	No. of patients against	*P*-value	FDR
Panc-AdenoCA	229	1781	5.07E-10	1.83E-08
Liver-HCC	271	1739	1.30E-09	2.34E-08
Prost-AdenoCA	105	1905	4.60E-06	4.14E-05
CNS-Oligo	18	1992	4.35E-06	4.14E-05
ColoRect-AdenoCA	52	1958	6.15E-04	4.43E-03
Eso-AdenoCa	91	1919	9.08E-04	5.45E-03
Kidney-RCC	119	1891	1.48E-03	7.60E-03
Stomach-AdenoCA	52	1958	4.92E-03	2.21E-02
CNS-Medullo	75	1935	5.69E-03	2.28E-02
Panc-Endocrine	52	1958	6.46E-03	2.32E-02

### Even low editing efficacy might be beneficial

**Figure 3.**
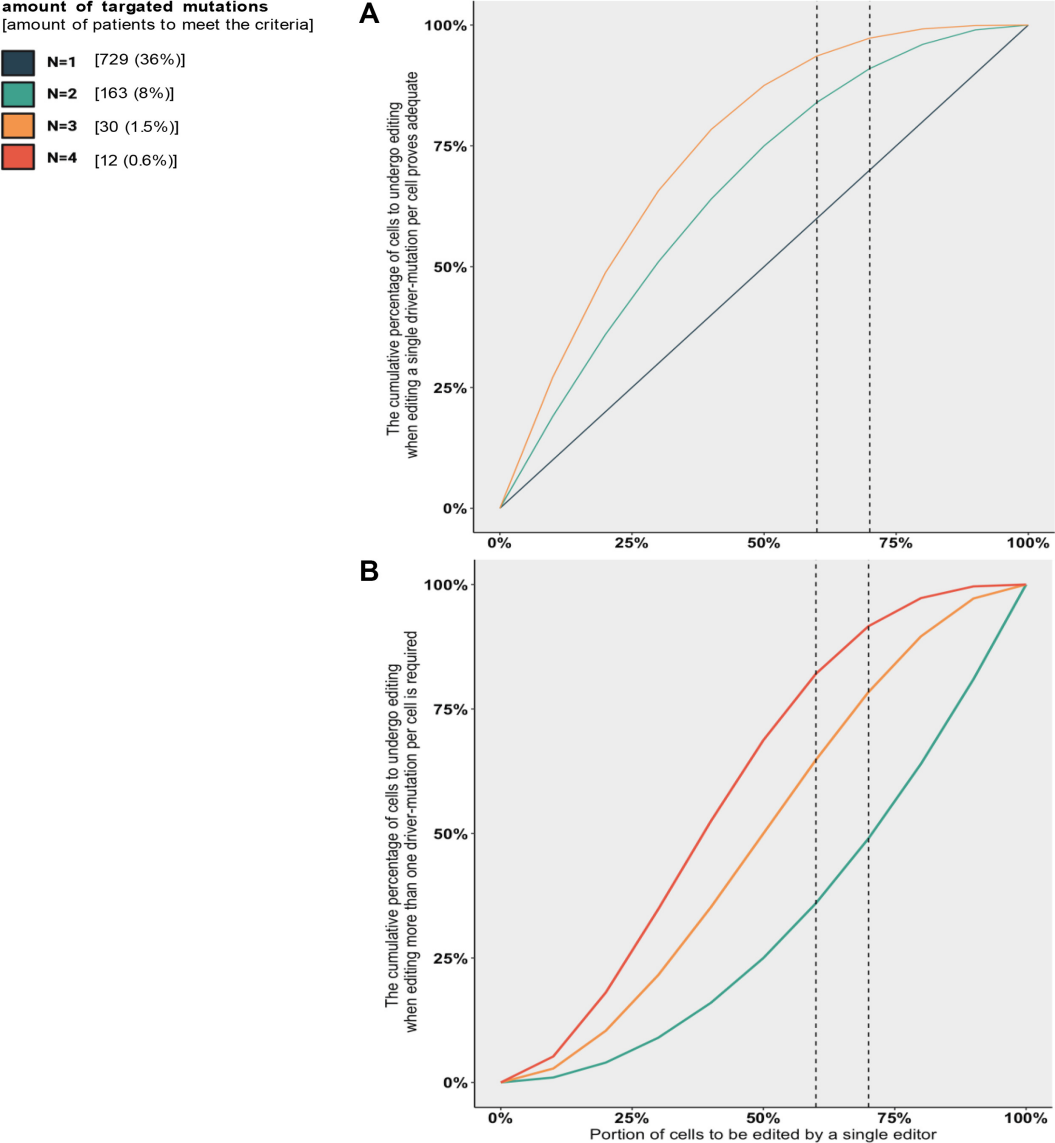
Calculation of the editing impact. The cumulative percentage of cells to undergo editing, presented by the therapeutic editing capability and the quantity of targeted variants. (**A**) When editing one driver mutation per cell is adequate. (**B**) When editing two driver mutations per cell is required.

Another aspect that necessitates further analysis pertains to the proportion of cells modified through therapeutic endogenous-ADAR. Based on current replicated studies, the efficacy of editing targeted cells is reported to range from 60% to 80% [34] when carefully engineering the optimal guides and incorporating precise chemical modifications. This gives rise to concerns in the context of cancer treatment, as tumor cells that evade editing may continue to progress. To quantify the potential impact of endogenous-ADAR therapy, we applied probabilistic arguments. For simplicity, we assume that all cancer cells exhibit the correctable driver mutations in that cancer type, and first calculated the aggregate proportion of cells that would be affected when targeting one, two, or three driver mutations simultaneously. We assume here a scenario in which (for multiple mutations), it is only the combination of all of these driver mutations that makes the cell malignant, and therefore successful editing of one driver mutation per cell suffices [40] (Fig. 3A). For instance, considering a lower bound of successful editing of just 60% and tumor cells presenting 1, 2, or 3 driver mutations, the fraction of treatable cells would be 60%, 84%, and 94%, respectively (Materials and methods).

While these calculations are based on theoretical assumptions that are likely to be more complicated in the real cancer environment, it is inferred that a substantial number of patients stand to gain from endogenous-ADAR treatment, even when a low editing threshold is employed. As technological advancements continue, the editing efficacy is likely to be high.

### Endogenous-ADAR correction of key cancer drivers: a few cases of considerable translational interest

**Figure 4.**
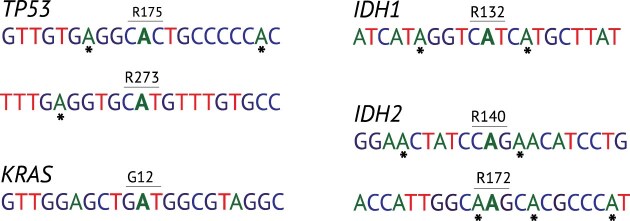
Targatable key cancer driver mutations. Illustration of six common cancer driver mutations in the *TP53*, *KRAS*, *IDH1*, and *IDH2* genes, amenable to endogenous-ADAR.

To demonstrate its clinical relevance in the context of cancer, we highlight here three major well known cancer drivers that are suitable for endogenous-ADAR out of many such examples that are provided in the various tables in Results. One obvious candidate is the tumor protein p53, encoded by the gene *TP53*, one of the most frequently mutated genes in cancer, acting as both tumor suppressor and oncogene and playing a crucial role in regulating cell cycle arrest and apoptosis [41]. *TP53* germline mutations are found in Li-Fraumeni syndrome [42], while somatic mutations are common in almost all cancer types. The most prevalent *TP53* variant, according to Cancer Genome Data (CGD) [43], is the R175H variant. This is a G > A missense variant that may serve as a good target for endogenous-ADAR in a variety of cancer types. Another frequently observed hotspot mutation in *TP53* is R273H, which is also a G > A missense variant. Both variants occur within the DNA-binding domain of the TP53 protein and are known to disrupt normal gene function [44, 45]. In the case of the first variant, there are two possible pathogenic bystander edits, whereas for the second variant, only one is observed (Fig. 4). No distant off-target hits were detected for either variant.

A second interesting example is isocitrate dehydrogenases (*IDH1* and *IDH2*), enzyme-encoding genes that are frequently mutated in gliomas, acute myeloid leukemia, cholangiocarcinoma, chondrosarcoma, and thyroid carcinoma [46]. Pathogenic mutations in IDH genes often occur at specific arginine residues (R132 for *IDH1*, R140 or R172 for *IDH2*), which are vital for isocitrate recognition. Consequently, these IDH variants have been extensively investigated as potential therapeutic targets for cancer treatment [47]. Interestingly, all three variants are G > A missense SNVs, rendering them suitable for endogenous ADAR. Predictions suggest that 2–3 bystander edits could be pathogenic upon editing (Fig. 4), with no detected off-target hits.

Finally, the *KRAS* proto-oncogene is a key player in cell signaling pathways governing cell growth and division. Its high occurrence of mutations across various cancers renders it a central subject in cancer research and therapy [48]. Among somatic mutations in KRAS, the most common one is G12D, which is also the most common KRAS mutation in carcinomas [49]. This is a G > A SNV, and our investigation reveals that no pathogenic bystander edits or distant off-targets are detected for this variant, making it a safe therapeutic target for endogenous-ADAR.

Several strategies have been purposed to treat patients whose tumors have this somatic variant. Though no clinical trial results are available to date, promising results were seen in preclinical models [50]. Thus, to date, no studies have assessed resistance mechanisms to G12D inhibitors, but resistance to treatment has been observed for KRAS–G12C inhibitors and may inform potential resistance mechanisms for G12D inhibitors. The approach we suggest here to edit the mutation might also lead to such resistance, evading the therapy. Yet, in the case of base editing, it is easier to retailor the guide to the patient once new mutations arise. This approach thus warrants further investigation.

## Discussion

This study presents a computational framework for exploring the capabilities of endogenous-ADAR editors in cancer risk reduction/prevention and cancer treatment. We first systematically studied the potential application scope of endogenous-ADAR editing to correct key mutations in childhood cancer predisposition syndromes, as well as high-penetrance germline mutations associated with cancer in adults. This approach could diminish the severity or impact of inherited cancer syndromes, changing the field of cancer risk. Complete resolution of germline mutations using endogenous-ADAR is still a distant goal, due to limited efficiency, but even partial correction might be beneficial in postponing the onset of cancer to a later age. Additionally, while RNA editing is transient, which can serve as an advantage when monitoring errors and unintended edits, it presents challenges in terms of patient compliance and burden. This is particularly true for cancer prevention which requires long-term therapy administration. Clearly, the benefits and drawbacks should be carefully weighed for each case. An inspiring example is of women carrying the mutated *BRCA1* or *BRCA2* genes, who are advised to undergo prophylactic removal of the uterus and ovaries. For these individuals, employing such a therapy to delay cancer development becomes crucial as it permits them to defer the surgery until after their fertile years.

Second, we have turned to study the extent by which endogenous-ADAR may potentially be harnessed for cancer treatment. For many of the patients studied in PCAWG, covering multiple cancer types, endogenous-ADAR can potentially target at least one identified driver mutation. Considering the successful application of lipid-nanoparticles (LNPs) as therapeutic agents in different cancer types [51], the prospects of delivering endogenous-ADAR to various cancers seem promising, as it requires the insertion of only a small payload containing a gRNA into the cell. While small-molecule targeted therapies may be preferable when available, we highlight the potential of endogenous-ADAR as a valuable treatment option in cases where other therapies are lacking or as an adjunct to existing treatments.

Off-target edits pose a serious safety concern. This is particularly true in the context of cancer prevention, where patients do not exhibit a phenotypic condition at the time of treatment, and any adverse outcomes resulting from off-target effects can undermine the potential benefits of the therapy. In contrast, when targeting somatic mutations within a developed tumor, the “price” for treating the disease may be more acceptable. Yet, since driver mutation may be present within noncancerous cells [52], the safety profile of the editor should be considered. A thorough investigation of potential off-target effects associated with the variants mentioned in the paper revealed a low probability of off-target occurrences. Variants with a sparse number of detected off-target sites may still serve as viable therapeutic targets, since the majority of genomic sites are noncoding, making unintended edits not clinically meaningful. Second, even if these off-target sites fall within coding regions, they are not necessarily expressed in the targeted tissue, underscoring another advantage of RNA editing over DNA editing. We also computed the potential bystander edits that may arise when the deaminase edits nucleotides in close proximity to the target site, and the number of those which are predicted to have a deleterious effect based on current available models.

An additional factor to consider is the degree of editing achieved per target. Reasonably, if the appropriate target is chosen, the mere editing of a subset of cells or RNA copies could still yield an effect in suppressing tumor growth. Moreover, combining multiple driver mutation targets could lead to improved results. A possible drawback might be that the treatment could lead to the clonal selection of cells not covered by the therapy. However, if new mutations arise, adjustments can be made to tailor the therapy accordingly. It is likely that the strength of the presented technique lies in slowing down disease progression rather than providing a complete cure for cancer. To delve deeper into these aspects and better grasp the potential of endogenous-ADAR in cancer-related applications, further *in vivo* research is necessary to confirm targeting specificity to specific cancer types and to minimize potential risk of off-target effects. Lastly, the native ADAR enzyme requires a specific motif, particularly necessitating the absence of a G nucleotide 5′ to the edited A. However, recent studies have identified strategies to overcome this constraint [53]; hence, it was not considered in our analysis. In the same manner, 5′-CAN-3′ sequences are generally disfavored and correction at these sites is challenging, but recent advancements propose methods to address this issue [54]. Secondary structures of the target transcript region can also impact proper binding of the gRNA to the desired region. All of these factors should be taken into account when carefully planning a guide for endogenous-ADAR therapy.

In this study, we investigated the prospects of endogenous-ADAR to target inherited variants associated with cancer risk in pediatric syndromes and adults with high-penetrance disease, as well as treating cancer by correcting driver mutations in tumors. Our analysis was conducted on external databases that might disproportionately represent specific populations in comparison to others. Yet, our findings portray a favorable and encouraging landscape of correctable mutations in cancer, pointing to the possibility of future leveraging the unique capabilities of endogenous-ADAR therapy towards clinical cancer risk/prevention and treatment outcomes.

## Supplementary Material

zcaf016_Supplemental_Files

## Data Availability

There are no primary data in the paper. The source code used to produce the results and analyses presented in this manuscript are available on a Zenodo at 10.5281/zenodo.14787914 https://doi.org/10.5281/zenodo.14778569. The ClinVar dataset, a publicly accessible repository of clinically relevant genomic variations managed by the National Center for Biotechnology Information (NCBI), was retrieved from UCSC, version November 2021, and can be accessed at https://hgdownload.soe.ucsc.edu/goldenpath/archive/hg38/clinvar/2021-11/. Proteome-wide missense variant effect predictions from the alphaMissense project were obtained from their community resource repository, which can be accessed at https://console.cloud.google.com/storage/browser/dm_alphamissense. The Memorial Sloan Kettering's SIGNAL db is an open-access database available at https://www.signaldb.org/. The PCWAG database was downloaded following an application for access to the ICGC Controlled Data.
