# Supplementary material for: A systematic evaluation of the therapeutic potential of endogenous-ADAR editors in cancer prevention and treatment
Source: NAR Cancer. 2025 May 6;7(2):zcaf016. doi: 10.1093/narcan/zcaf016 (PMC12053386; doi:10.1093/narcan/zcaf016)
Supplement: zcaf016_Supplemental_Files [file zcaf016_supplemental_files.zip › Supplementary file.docx]

**Supportive information**

All supplementary tables can be downloaded from this GitHub link:

<https://github.com/arieldadush/BE-in-Cancer-Prevention-and-Treatment>

**Table S1:** Cancer predisposition genes (CPGs) that are tested in a panel-test offered by ‘PreventionGenetics’ for pediatric genetic testing (https://www.ncbi.nlm.nih.gov/gtr/tests/562503/) and their associated clinical condition.

|  | **Gene** | **Conditions/Phenotypes** |
| --- | --- | --- |
| 1 | [*ALK* (2p23.2-23.1)](https://www.ncbi.nlm.nih.gov/gtr/genes/238/) | [Neuroblastoma, susceptibility to, 3](https://www.ncbi.nlm.nih.gov/gtr/conditions/C2751681/) |
| 2 | [*ANKRD26* (10p12.1)](https://www.ncbi.nlm.nih.gov/gtr/genes/22852/) | [Thrombocytopenia 2](https://www.ncbi.nlm.nih.gov/gtr/conditions/C1861185/) |
| 3 | [*APC* (5q22.2)](https://www.ncbi.nlm.nih.gov/gtr/genes/324/) | [Desmoid disease, hereditary](https://www.ncbi.nlm.nih.gov/gtr/conditions/C1851124/) |
|  |  | [Familial adenomatous polyposis 1](https://www.ncbi.nlm.nih.gov/gtr/conditions/C2713442/) |
| 4 | [*ATM* (11q22.3)](https://www.ncbi.nlm.nih.gov/gtr/genes/472/) | [Ataxia-telangiectasia syndrome](https://www.ncbi.nlm.nih.gov/gtr/conditions/C0004135/) |
| 5 | [*AXIN2* (17q24.1)](https://www.ncbi.nlm.nih.gov/gtr/genes/8313/) | [Oligodontia-cancer predisposition syndrome](https://www.ncbi.nlm.nih.gov/gtr/conditions/C1837750/) |
| 6 | [*BAP1* (3p21.1)](https://www.ncbi.nlm.nih.gov/gtr/genes/8314/) | [BAP1-related tumor predisposition syndrome](https://www.ncbi.nlm.nih.gov/gtr/conditions/C3280492/) |
| 7 | [*BLM* (15q26.1)](https://www.ncbi.nlm.nih.gov/gtr/genes/641/) | [Bloom syndrome](https://www.ncbi.nlm.nih.gov/gtr/conditions/C0005859/) |
| 8 | [*BMPR1A* (10q23.2)](https://www.ncbi.nlm.nih.gov/gtr/genes/657/) | [Generalized juvenile polyposis/juvenile polyposis coli](https://www.ncbi.nlm.nih.gov/gtr/conditions/C1868081/) |
|  |  | [Polyposis syndrome, hereditary mixed, 2](https://www.ncbi.nlm.nih.gov/gtr/conditions/C1864730/) |
| 9 | [*CDC73* (1q31.2)](https://www.ncbi.nlm.nih.gov/gtr/genes/79577/) | [Parathyroid carcinoma](https://www.ncbi.nlm.nih.gov/gtr/conditions/C0687150/) |
| 10 | [*CDKN1C* (11p15.4)](https://www.ncbi.nlm.nih.gov/gtr/genes/1028/) | [Beckwith-Wiedemann syndrome](https://www.ncbi.nlm.nih.gov/gtr/conditions/C0004903/) |
| 11 | *C*[*EBPA* (19q13.11)](https://www.ncbi.nlm.nih.gov/gtr/genes/1050/) | [Acute myeloid leukemia](https://www.ncbi.nlm.nih.gov/gtr/conditions/C0023467/) |
| 12 | [*DDX41* (5q35.3)](https://www.ncbi.nlm.nih.gov/gtr/genes/51428/) | [DDX41-related hematologic malignancy predisposition syndrome](https://www.ncbi.nlm.nih.gov/gtr/conditions/C4225174/) |
| 13 | [*DICER1* (14q32.13)](https://www.ncbi.nlm.nih.gov/gtr/genes/23405/) | [Euthyroid goiter](https://www.ncbi.nlm.nih.gov/gtr/conditions/C0302859/) |
|  |  | [Rhabdomyosarcoma, embryonal, 2](https://www.ncbi.nlm.nih.gov/gtr/conditions/C1867234/) |
|  |  | [DICER1 syndrome](https://www.ncbi.nlm.nih.gov/gtr/conditions/C3839822/) |
| 14 | [*DIS3L2* (2q37.1)](https://www.ncbi.nlm.nih.gov/gtr/genes/129563/) | [Perlman syndrome](https://www.ncbi.nlm.nih.gov/gtr/conditions/C0796113/) |
| 15 | [*EPCAM* (2p21)](https://www.ncbi.nlm.nih.gov/gtr/genes/4072/) | [Lynch syndrome 8](https://www.ncbi.nlm.nih.gov/gtr/conditions/C2750471/) |
| 16 | [*ETV6* (12p13.2)](https://www.ncbi.nlm.nih.gov/gtr/genes/2120/) | [Acute myeloid leukemia](https://www.ncbi.nlm.nih.gov/gtr/conditions/C0023467/) |
|  |  | [Thrombocytopenia 5](https://www.ncbi.nlm.nih.gov/gtr/conditions/C4015537/) |
| 17 | [*EXT1* (8q24.11)](https://www.ncbi.nlm.nih.gov/gtr/genes/2131/) | [Multiple congenital exostoses](https://www.ncbi.nlm.nih.gov/gtr/conditions/C0015306/), type 1 |
| 18 | [EXT2 (11p11.2)](https://www.ncbi.nlm.nih.gov/gtr/genes/2132/) | [Exostoses, multiple, type 2](https://www.ncbi.nlm.nih.gov/gtr/conditions/C1851413/) |
| 19 | [*FH* (1q43)](https://www.ncbi.nlm.nih.gov/gtr/genes/2271/) | [Hereditary leiomyomatosis and renal cell cancer](https://www.ncbi.nlm.nih.gov/gtr/conditions/C1708350/) |
| 20 | [*GATA2* (3q21.3)](https://www.ncbi.nlm.nih.gov/gtr/genes/2624/) | [Acute myeloid leukemia](https://www.ncbi.nlm.nih.gov/gtr/conditions/C0023467/) |
| 21 | [*GPC3* (Xq26.2)](https://www.ncbi.nlm.nih.gov/gtr/genes/2719/) | [Wilms tumor 1](https://www.ncbi.nlm.nih.gov/gtr/conditions/CN033288/) |
|  |  | [Simpson-Golabi-Behmel syndrome type 1](https://www.ncbi.nlm.nih.gov/gtr/conditions/C0796154/) |
| 22 | [*HRAS* (11p15.5)](https://www.ncbi.nlm.nih.gov/gtr/genes/3265/) | [Costello syndrome](https://www.ncbi.nlm.nih.gov/gtr/conditions/C0587248/) |
| 23 | [*KIF1B* (1p36.22)](https://www.ncbi.nlm.nih.gov/gtr/genes/23095/) | [Neuroblastoma](https://www.ncbi.nlm.nih.gov/gtr/conditions/C0027819/) |
|  |  | [Pheochromocytoma](https://www.ncbi.nlm.nih.gov/gtr/conditions/C0031511/) |
| 24 | [*MAX* (14q23.3)](https://www.ncbi.nlm.nih.gov/gtr/genes/4149/) | [Pheochromocytoma](https://www.ncbi.nlm.nih.gov/gtr/conditions/C0031511/) |
| 25 | [*MEN1* (11q13.1)](https://www.ncbi.nlm.nih.gov/gtr/genes/4221/) | [Multiple endocrine neoplasia, type 1](https://www.ncbi.nlm.nih.gov/gtr/conditions/C0025267/) |
| 26 | [*MLH1* (3p22.2)](https://www.ncbi.nlm.nih.gov/gtr/genes/4292/) | [Turcot syndrome](https://www.ncbi.nlm.nih.gov/gtr/conditions/C5399763/) |
|  |  | [Muir-Torré syndrome](https://www.ncbi.nlm.nih.gov/gtr/conditions/C1321489/) |
|  |  | [Colorectal cancer, hereditary nonpolyposis, (Lynch syndrome) type 2](https://www.ncbi.nlm.nih.gov/gtr/conditions/C1333991/) |
| 27 | [*MSH2* (2p21-16.3)](https://www.ncbi.nlm.nih.gov/gtr/genes/4436/) | [Lynch syndrome type 1](https://www.ncbi.nlm.nih.gov/gtr/conditions/C2936783/) |
|  |  | [Turcot syndrome](https://www.ncbi.nlm.nih.gov/gtr/conditions/C5399763/) |
|  |  | [Muir-Torré syndrome](https://www.ncbi.nlm.nih.gov/gtr/conditions/C1321489/) |
| 28 | [*MSH6* (2p16.3)](https://www.ncbi.nlm.nih.gov/gtr/genes/2956/) | [Turcot syndrome](https://www.ncbi.nlm.nih.gov/gtr/conditions/C5399763/) |
|  |  | [Lynch syndrome type 5](https://www.ncbi.nlm.nih.gov/gtr/conditions/C1833477/) |
| 29 | [*NBN* (8q21.3)](https://www.ncbi.nlm.nih.gov/gtr/genes/4683/) | [Microcephaly, normal intelligence and immunodeficiency](https://www.ncbi.nlm.nih.gov/gtr/conditions/C0398791/) (Nijmegen breakage syndrome) |
|  |  | [Acute lymphoid leukemia](https://www.ncbi.nlm.nih.gov/gtr/conditions/C0023449/) |
| 30 | [*NF1* (17q11.2)](https://www.ncbi.nlm.nih.gov/gtr/genes/4763/) | [Neurofibromatosis, familial spinal](https://www.ncbi.nlm.nih.gov/gtr/conditions/C1834235/) |
|  |  | [Neurofibromatosis, type 1](https://www.ncbi.nlm.nih.gov/gtr/conditions/C0027831/) |
|  |  | [Neurofibromatosis-Noonan syndrome](https://www.ncbi.nlm.nih.gov/gtr/conditions/C2931482/) |
| 31 | [*NF2* (22q12.2)](https://www.ncbi.nlm.nih.gov/gtr/genes/4771/) | [Familial meningioma](https://www.ncbi.nlm.nih.gov/gtr/conditions/C3551915/) |
|  |  | [Neurofibromatosis, type 2](https://www.ncbi.nlm.nih.gov/gtr/conditions/C0027832/) |
|  |  | [Schwannomatosis 1](https://www.ncbi.nlm.nih.gov/gtr/conditions/C4048809/) |
| 32 | [*PHOX2B* (4p13)](https://www.ncbi.nlm.nih.gov/gtr/genes/8929/) | [Neuroblastoma, susceptibility to, 2](https://www.ncbi.nlm.nih.gov/gtr/conditions/C2751682/) |
| 33 | [*PMS2* (7p22.1)](https://www.ncbi.nlm.nih.gov/gtr/genes/5395/) | [Turcot syndrome](https://www.ncbi.nlm.nih.gov/gtr/conditions/C5399763/) |
|  |  | [Lynch syndrome type 4](https://www.ncbi.nlm.nih.gov/gtr/conditions/C1838333/) |
| 34 | [*POT1* (7q31.33)](https://www.ncbi.nlm.nih.gov/gtr/genes/25913/) | [Melanoma, cutaneous malignant, susceptibility to, 10](https://www.ncbi.nlm.nih.gov/gtr/conditions/C4014476/) |
|  |  | [Glioma susceptibility 9](https://www.ncbi.nlm.nih.gov/gtr/conditions/C4225278/) |
| 35 | [*PRKAR1A* (17q24.2)](https://www.ncbi.nlm.nih.gov/gtr/genes/5573/) | [Carney complex, type 1](https://www.ncbi.nlm.nih.gov/gtr/conditions/C2607929/) |
|  |  | [Pigmented nodular adrenocortical disease, primary, 1](https://www.ncbi.nlm.nih.gov/gtr/conditions/C1864846/) |
|  |  | [Familial atrial myxoma](https://www.ncbi.nlm.nih.gov/gtr/conditions/C2931787/) |
| 36 | [*PTCH1* (9q22.32)](https://www.ncbi.nlm.nih.gov/gtr/genes/5727/) | [Gorlin syndrome](https://www.ncbi.nlm.nih.gov/gtr/conditions/C0004779/) |
| 37 | [*PTCH2* (1p34.1)](https://www.ncbi.nlm.nih.gov/gtr/genes/8643/) | [Gorlin syndrome](https://www.ncbi.nlm.nih.gov/gtr/conditions/C0004779/) |
| 38 | [*PTEN* (10q23.31)](https://www.ncbi.nlm.nih.gov/gtr/genes/5728/) | [Familial meningioma](https://www.ncbi.nlm.nih.gov/gtr/conditions/C3551915/) |
|  |  | [Cowden syndrome 1](https://www.ncbi.nlm.nih.gov/gtr/conditions/CN072330/) |
| 39 | [*RB1* (13q14.2)](https://www.ncbi.nlm.nih.gov/gtr/genes/5925/) | [Retinoblastoma](https://www.ncbi.nlm.nih.gov/gtr/conditions/C0035335/) |
| 40 | [*REST* (4q12)](https://www.ncbi.nlm.nih.gov/gtr/genes/5978/) | [Wilms tumor 6](https://www.ncbi.nlm.nih.gov/gtr/conditions/C3891301/) |
| 41 | [*RET* (10q11.21)](https://www.ncbi.nlm.nih.gov/gtr/genes/5979/) | [Multiple endocrine neoplasia, type 2b](https://www.ncbi.nlm.nih.gov/gtr/conditions/C0025269/) |
|  |  | [Pheochromocytoma](https://www.ncbi.nlm.nih.gov/gtr/conditions/C0031511/) |
|  |  | [Familial medullary thyroid carcinoma](https://www.ncbi.nlm.nih.gov/gtr/conditions/C1833921/) |
|  |  | [Multiple endocrine neoplasia, type 2a](https://www.ncbi.nlm.nih.gov/gtr/conditions/C0025268/) |
| 42 | [*RUNX1* (21q22.12)](https://www.ncbi.nlm.nih.gov/gtr/genes/861/) | [Hereditary thrombocytopenia and hematological cancer predisposition syndrome associated with RUNX1](https://www.ncbi.nlm.nih.gov/gtr/conditions/C1832388/) |
|  |  | [Acute myeloid leukemia](https://www.ncbi.nlm.nih.gov/gtr/conditions/C0023467/) |
| 43 | [*SAMD9L* (7q21.2)](https://www.ncbi.nlm.nih.gov/gtr/genes/219285/) | [Ataxia-pancytopenia syndrome](https://www.ncbi.nlm.nih.gov/gtr/conditions/C1327919/) |
| 44 | [*SDHA* (5p15.33)](https://www.ncbi.nlm.nih.gov/gtr/genes/6389/) | [Paragangliomas 5](https://www.ncbi.nlm.nih.gov/gtr/conditions/C3279992/) |
| 45 | [*SDHAF2* (11q12.2)](https://www.ncbi.nlm.nih.gov/gtr/genes/54949/) | [Paragangliomas 2](https://www.ncbi.nlm.nih.gov/gtr/conditions/C1866552/) |
| 46 | [*SDHB* (1p36.13)](https://www.ncbi.nlm.nih.gov/gtr/genes/6390/) | [Paragangliomas 4](https://www.ncbi.nlm.nih.gov/gtr/conditions/C1861848/) |
|  |  | [Pheochromocytoma](https://www.ncbi.nlm.nih.gov/gtr/conditions/C0031511/) |
|  |  | Cowden syndrome |
| 47 | [*SDHC* (1q23.3)](https://www.ncbi.nlm.nih.gov/gtr/genes/6391/) | [Paragangliomas 3](https://www.ncbi.nlm.nih.gov/gtr/conditions/C1854336/) |
| 48 | [*SDHD* (11q23.1)](https://www.ncbi.nlm.nih.gov/gtr/genes/6392/) | [Pheochromocytoma](https://www.ncbi.nlm.nih.gov/gtr/conditions/C0031511/) |
|  |  | [Carcinoid tumor of intestine](https://www.ncbi.nlm.nih.gov/gtr/conditions/C0349535/) |
|  |  | [Paragangliomas 1](https://www.ncbi.nlm.nih.gov/gtr/conditions/C3494181/) |
|  |  | Cowden syndrome |
| 49 | [*SMAD4* (18q21.2)](https://www.ncbi.nlm.nih.gov/gtr/genes/4089/) | [Generalized juvenile polyposis/juvenile polyposis coli](https://www.ncbi.nlm.nih.gov/gtr/conditions/C1868081/) |
|  |  | [Juvenile polyposis/hereditary hemorrhagic telangiectasia syndrome](https://www.ncbi.nlm.nih.gov/gtr/conditions/C1832942/) |
| 50 | [*SMARCA4* (19p13.2)](https://www.ncbi.nlm.nih.gov/gtr/genes/6597/) | [Rhabdoid tumor predisposition syndrome 2](https://www.ncbi.nlm.nih.gov/gtr/conditions/C2750074/) |
| 51 | [*SMARCB1* (22q11.23)](https://www.ncbi.nlm.nih.gov/gtr/genes/6598/) | [Schwannomatosis 1](https://www.ncbi.nlm.nih.gov/gtr/conditions/C4048809/) |
| 52 | [*SMARCE1* (17q21.2)](https://www.ncbi.nlm.nih.gov/gtr/genes/6605/) | [Familial meningioma](https://www.ncbi.nlm.nih.gov/gtr/conditions/C3551915/) |
| 53 | [*SRP72* (4q12)](https://www.ncbi.nlm.nih.gov/gtr/genes/6731/) | [Autosomal dominant aplasia and myelodysplasia](https://www.ncbi.nlm.nih.gov/gtr/conditions/C3808553/) |
| 54 | [*STK11* (19p13.3)](https://www.ncbi.nlm.nih.gov/gtr/genes/6794/) | [Peutz-Jeghers syndrome](https://www.ncbi.nlm.nih.gov/gtr/conditions/C0031269/) |
| 55 | [*SUFU* (10q24.32)](https://www.ncbi.nlm.nih.gov/gtr/genes/51684/) | [Gorlin syndrome](https://www.ncbi.nlm.nih.gov/gtr/conditions/C0004779/) |
|  |  | [Familial meningioma](https://www.ncbi.nlm.nih.gov/gtr/conditions/C3551915/) |
| 56 | [*TERC* (3q26.2)](https://www.ncbi.nlm.nih.gov/gtr/genes/7012/) | [Dyskeratosis congenita, autosomal dominant 1](https://www.ncbi.nlm.nih.gov/gtr/conditions/C4551974/) |
| 57 | [*TERT* (5p15.33)](https://www.ncbi.nlm.nih.gov/gtr/genes/7015/) | [Acute myeloid leukemia](https://www.ncbi.nlm.nih.gov/gtr/conditions/C0023467/) |
|  |  | [Dyskeratosis congenita, autosomal dominant 2](https://www.ncbi.nlm.nih.gov/gtr/conditions/C3151443/) |
| 58 | [*TMEM127* (2q11.2)](https://www.ncbi.nlm.nih.gov/gtr/genes/55654/) | [Pheochromocytoma](https://www.ncbi.nlm.nih.gov/gtr/conditions/C0031511/) |
| 59 | [*TP53* (17p13.1)](https://www.ncbi.nlm.nih.gov/gtr/genes/7157/) | [Adrenocortical carcinoma, hereditary](https://www.ncbi.nlm.nih.gov/gtr/conditions/C1859972/) |
|  |  | [Li-Fraumeni syndrome 1](https://www.ncbi.nlm.nih.gov/gtr/conditions/C1835398/) |
|  |  | [Choroid plexus papilloma](https://www.ncbi.nlm.nih.gov/gtr/conditions/C0205770/) |
|  |  | [Basal cell carcinoma, susceptibility to, 7](https://www.ncbi.nlm.nih.gov/gtr/conditions/C3553606/) |
| 60 | [*TRIP13* (5p15.33)](https://www.ncbi.nlm.nih.gov/gtr/genes/9319/) | [Mosaic variegated aneuploidy syndrome 3](https://www.ncbi.nlm.nih.gov/gtr/conditions/C4539839/) |
| 61 | [*TSC1* (9q34.13)](https://www.ncbi.nlm.nih.gov/gtr/genes/7248/) | [Tuberous sclerosis 1](https://www.ncbi.nlm.nih.gov/gtr/conditions/C1854465/) |
| 62 | [*TSC2* (16p13.3)](https://www.ncbi.nlm.nih.gov/gtr/genes/7249/) | [Tuberous sclerosis 2](https://www.ncbi.nlm.nih.gov/gtr/conditions/C1860707/) |
| 63 | [*VHL* (3p25.3)](https://www.ncbi.nlm.nih.gov/gtr/genes/7428/) | [Pheochromocytoma](https://www.ncbi.nlm.nih.gov/gtr/conditions/C0031511/) |
|  |  | [von Hippel-Lindau syndrome](https://www.ncbi.nlm.nih.gov/gtr/conditions/C0019562/) |
| 64 | [*WT1* (11p13)](https://www.ncbi.nlm.nih.gov/gtr/genes/7490/) | [Wilms tumor 1](https://www.ncbi.nlm.nih.gov/gtr/conditions/CN033288/) |

**Table S2** : **All ClinVar pathogenic SNVs located in cancer predisposition genes that are suitable for endogenous-ADAR.**

The table Includes our added data on bystander edits, off-target hits and ADAR motif information.

orig_name – ClinVar variant full name

location – genomic position

gene – gene

strand – DNA strand [ +/- ]

mutation – SNV mismatch type

mol_conseq – molecular consequence

clin_significance – clinical significance, according to ClinVar

phenotype_list – phenotypes associated with this variant, according to ClinVar

num_submit – numbers of submitters

last_date_eval – last evaluation date by ClinVar

gnomAD_freq – Variant frequency according to gnomAD

ADAR_motif – existence of ADAR motif [ yes/no]

gRNA_41bases – a sequence of 41 bases surrounding the variant (20 from each side) that represents the guide binding area

off_targets_85_min_identity – number of genomic off-target hits with at least 85% identity

count_bystander_edits – number of bases of the same type as the variant, within the 20 bases window surrounding the variant (10 from each side)

count_ pathogenic_bystander_edits - number of bases of the same type, within the 20 bases window surrounding the variant (10 from each side), that are predicted to be pathogenic upon editing. Based on the alphaMissense prediction tool.
